# The Repeatability Assessment of Three-Dimensional Capsule-Intraocular Lens Complex Measurements by Means of High-Speed Swept-Source Optical Coherence Tomography

**DOI:** 10.1371/journal.pone.0142556

**Published:** 2015-11-23

**Authors:** Xixia Ding, Qinmei Wang, Pingjun Chang, Jin Li, Giacomo Savini, Jinhai Huang, Shenghai Huang, Yinying Zhao, Na Liao, Lei Lin, Xiaoyu Yu, Yun-e Zhao

**Affiliations:** 1 School of Optometry and Ophthalmology and Eye Hospital, Wenzhou Medical University, Wenzhou, Zhejiang, China; 2 Key Laboratory of Vision Science, Ministry of Health P.R. China, Wenzhou, Zhejiang, China; 3 G.B. Bietti Foundation IRCCS, Rome, Italy; Tufts University, UNITED STATES

## Abstract

**Purpose:**

To rebuild the three-dimensional (3-D) model of the anterior segment by high-speed swept-source optical coherence tomography (SSOCT) and evaluate the repeatability of measurement for the parameters of capsule-intraocular lens (C-IOL) complex.

**Methods:**

Twenty-two pseudophakic eyes from 22 patients were enrolled. Three continuous SSOCT measurements were performed in all eyes and the tomograms obtained were used for 3-D reconstruction. The output data were used to evaluate the measurement repeatability. The parameters included postoperative aqueous depth (PAD), the area and diameter of the anterior capsule opening (Area and D), IOL tilt (IOL-T), horizontal, vertical, and space decentration of the IOL, anterior capsule opening, and IOL-anterior capsule opening.

**Results:**

PAD, IOL-T, Area, D, and all decentration measurements showed high repeatability. Repeated measure analysis showed there was no statistically significant difference among the three continuous measurements (all P > .05). Pearson correlation analysis showed high correlation between each pair of them (all r >0.90, P<0.001). ICCs were all more than 0.9 for all parameters. The 95% LoAs of all parameters were narrow for comparison of three measurements, which showed high repeatability for three measurements.

**Conclusion:**

SSOCT is available to be a new method for the 3-D measurement of C-IOL complex after cataract surgery. This method presented high repeatability in measuring the parameters of the C-IOL complex.

## Introduction

A significant interaction between the capsular bag and the intraocular lens (IOL) is maintained for a long time after cataract surgery.[1, 2] Any change of the capsule-IOL (C-IOL) complex can have important consequences on the postoperative IOL stability and subsequent refraction,[1, 3] and posterior capsule opacity (PCO).[2, 4–7] Thanks to the technological advances of IOL designs and IOL power calculation, cataract surgery can now be considered a refractive procedure.[8] However, premium IOLs, including aspherical, toric, and multifocal IOLs, are more sensitive to the position in the capsule bag. A small amount of IOL movement will slow down the performance of these IOLs. Thus, the precise measurement and objective evaluation of the C-IOL complex might provide the basis for IOL design improvement, which may lead to better clinical outcomes in the future. Previous studies have focused on the C-IOL complex in vivo using different devices, including slitlamp,[2] Scheimpflug videophotography (EAS-1000), [1] and optical coherence tomography (OCT).[9–11]

OCT is a non-contact and high-resolution technology, which in a few seconds can provide anterior segment images that are easy to be interpreted. For these reasons, OCT is an optimal method to observe the capsule-IOL complex in vivo. Several types of OCT have been used to observe the anterior segment of eye. However, the Visante (Carl Zeiss Meditec, Dublin, CA) and RTvue OCT (Optovue Inc., CA, U.S.), widely used in clinic, are unable to make a full and high definition image of the capsule-IOL complex.[9, 12] Several custom-built OCT instruments were reported to capture high-quality tomograms for the whole complex, but all views shown were 2-dimensional (2-D).[10, 11, 13] Additionally, previous studies showed that 3-dimensional (3-D) quantitative analysis of full anterior segment has been available using some custom-built OCT devices.[14–16] However, there has been no study that reported the 3-D capsule reconstruction with OCT.

The purpose of the current study was to realize the 3-D reconstruction of the capsule-IOL complex by means of SSOCT and to assess the repeatability of this new method.

## Methods

**Fig 1:**
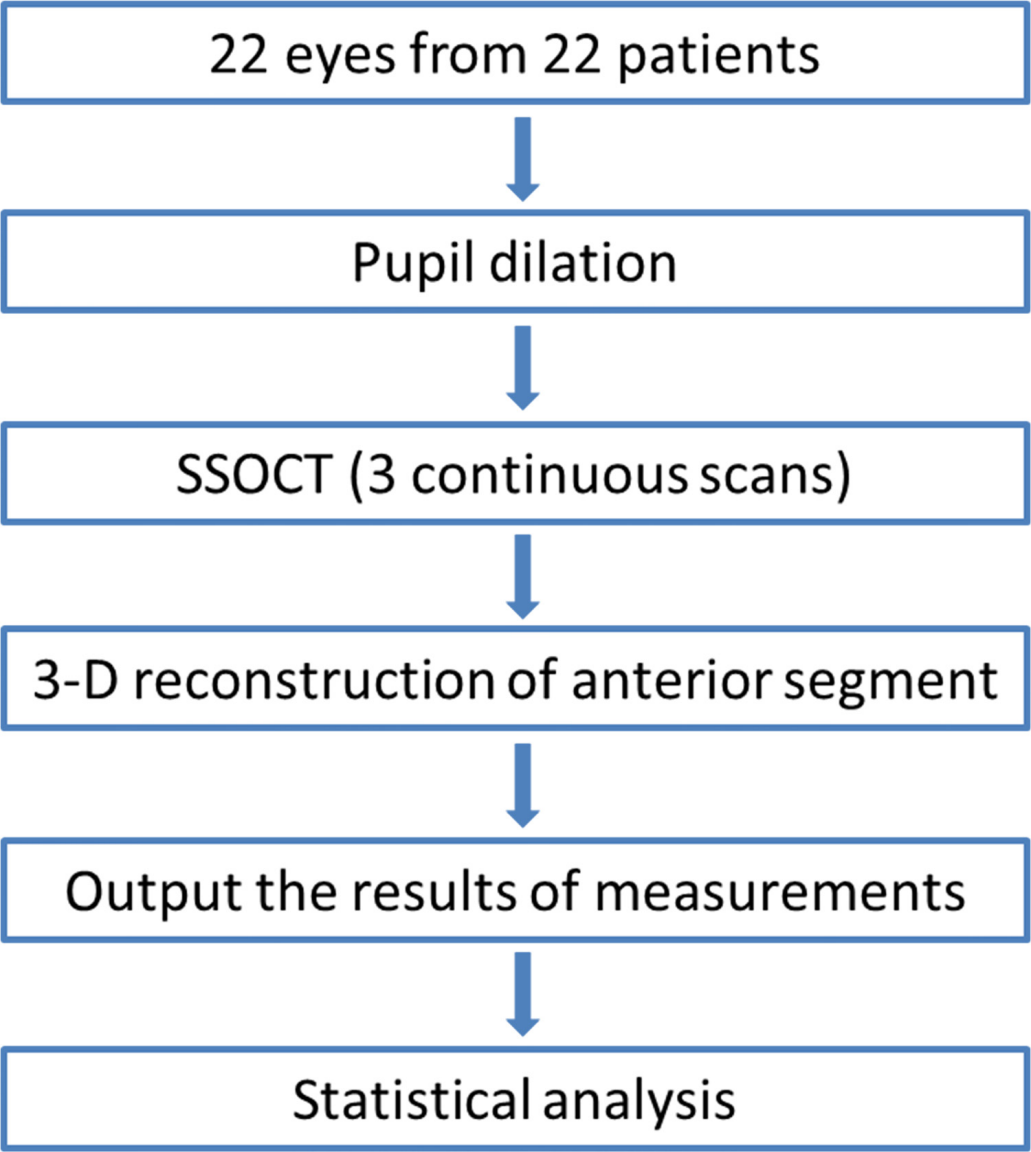
The study CONSORT flowchart.

A CONSORT flowchart was shown in Fig 1.

### Patients

In this study, we enrolled 22 patients (22 eyes) who had undergone phacoemulsification and IOL implantation from 6 months to 2 years before to assess the repeatability of 3-D reconstruction measurements. One eye was chosen randomly when both eyes were met the inclusion standards. In all cases, a one-piece hydrophobic, square-edged IOL (Acrysof SN60WF, Alcon, Fort Worth, TX) had been implanted.

This study was conducted at the Eye Hospital of Wenzhou Medical University, China, from Oct 1st, 2013 to Dec 31st, 2013. The research protocol was in accordance with the Declaration of Helsinki and was approved by the Office of Research Ethics, Eye Hospital of Wenzhou Medical University. Written informed consent was obtained from each subject.

The exclusion criteria were as follows: 1) less than 40 years old; 2) axial length ≥ 26 mm; 3) eye diseases including any corneal pathology, uveitis, and glaucoma; 4) previous intraocular surgery other than phacoemulsification; 5) any intraoperative or postoperative complication; 6) dilated pupil diameter < 7 mm; 7) failure to take examinations. Before inclusion in our study, all eyes underwent a complete ophthalmic examination including refraction, slit-lamp microscopy, noncontact tonometry, optical biometry (IOLMaster 5.0, Carl Zeiss, Germany), and ophthalmoscopy after pupil dilation.

### Instrument and Analysis Software

**Fig 2:**
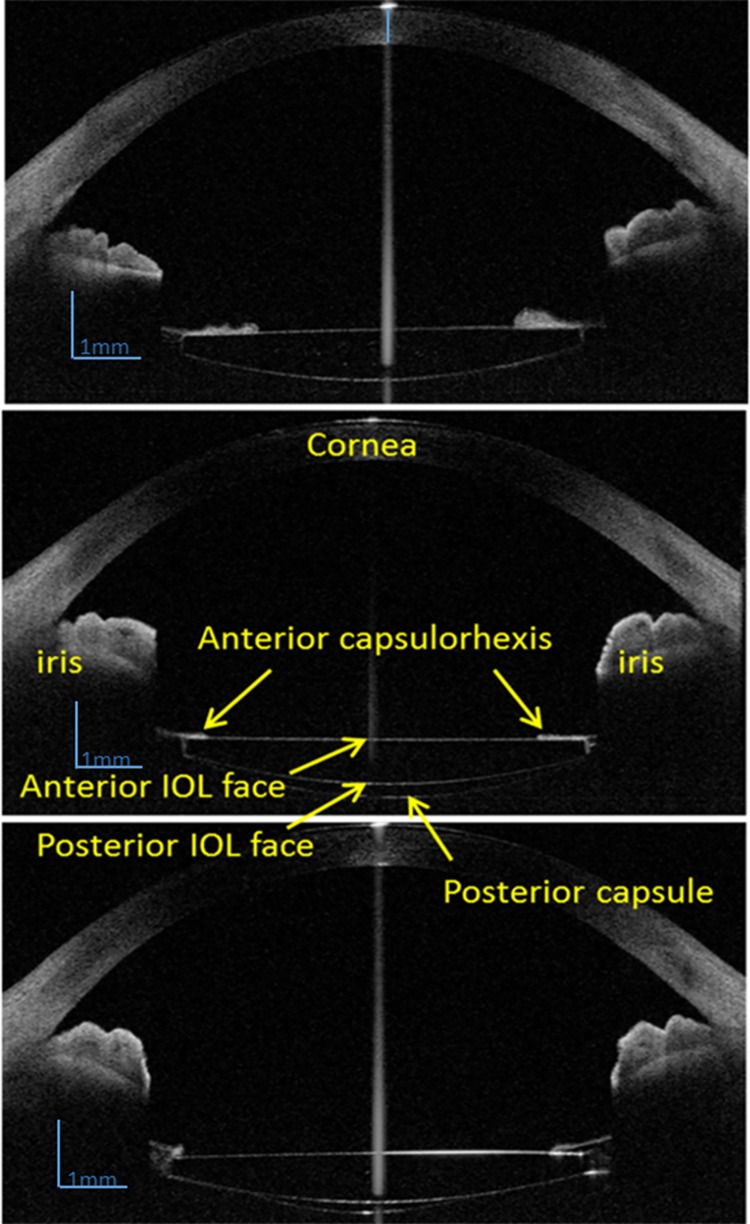
The anterior segment images of high-speed swept-source optical coherence tomography (SSOCT).

The SSOCT (Casia SS-1000, Tomey, Nagoya, Japan), a form of Fourier-domain OCT, is a commercially available swept-source OCT with a swept-source laser wavelength of 1310 nm. In the 3-D mode, there are 5 different scanning types, including anterior segment (standard), angle analysis, angle HD, bleb, and corneal map. In this study, the radial 3-D angle analysis auto-off mode, which was handled manually rather than automatically, was chosen to obtain the anterior segment images. It scans very fast with a speed of 30,000 A scans/second and 512 lines A scan per image sampling. Each 3-D radial scanning takes only 2.4 seconds and 128 cross-sectional images of anterior segment are captured. The high-quality SSOCT images are shown in Fig 2. Parameters of SSOCT in this mode are shown in **Table 1**.

**Table 1:** Parameters of swept source optical coherence tomography (SSOCT) in the radial three-dimensional (3-D) scan mode.

Parameters		
**Light-source unit**		
	Type of light source	Swept source Laser
	Wavelength	1310 nm
**Resolution**		
	Axial (Depth)	10μm or less (in tissue)
	Transverse	30μm or less (in tissue)
**Scan range**		
	Depth	6 mm
	Transverse	Radial scan diameter of 16mm

**Fig 3:**
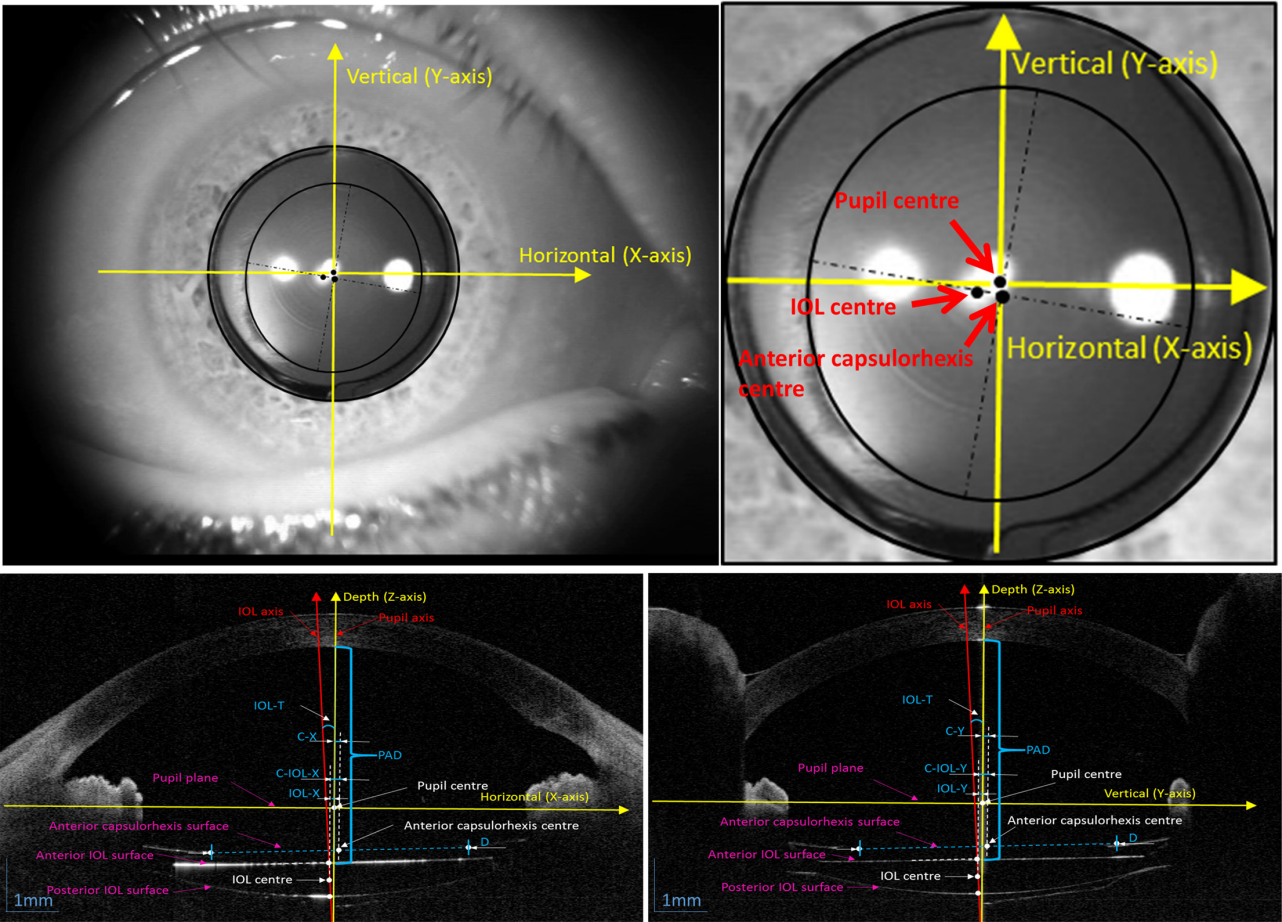
Definition for all parameters in different cross-sectional views. PAD = Postoperative aqueous depth; IOL = intraocular lens; IOL-T = intraocular lens tilt; D = Anterior capsulorhexis diameter; IOL-X = IOL decentration in the x-axis; IOL-Y = IOL decentration in the y-axis; C-X = Anterior capsulorhexis decentration in the x-axis; C-Y = Anterior capsulorhexis decentration in the y-axis; C-IOL-X = Anterior capsulorhexis-IOL decentration in the x-axis; C-IOL-Y = Anterior capsulorhexis-IOL decentration in the y-axis.

**Fig 4:**
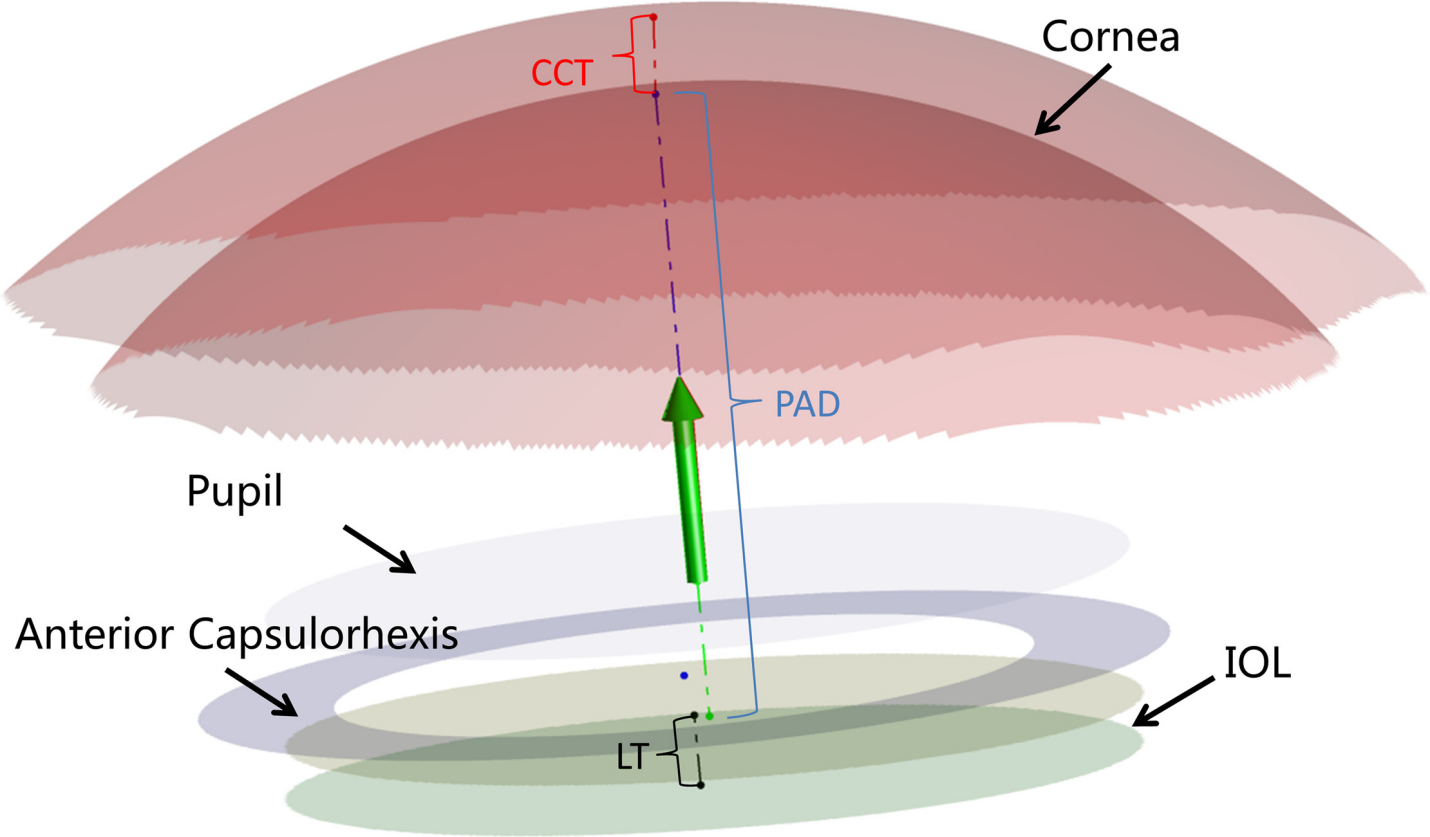
Three-dimensional constructed model of the anterior segment. This image clearly showed the whole anterior segment of the eye, including cornea, anterior chamber, pupil, anterior capsulorhexis, and intraocular lens (IOL). CCT = centre corneal thickness; PAD = postoperative aqueous depth; LT = lens thickness.

The 3-D reconstruction and analysis software was developed by the OCT research team in Eye Hospital of Wenzhou Medical University. It is a special type of software which is match up with the SSOCT images. Firstly, 128 images were extracted from the video, which was exported from built-in software of SSOCT. Although the semi-automatic algorithm was achieved, the segment results still required individual inspection. So the 16 cross-sectional images with equal intervals from all sections were chosen for anterior segment reconstruction without significant loss of accuracy. Then the work and computation complexity could be significantly reduced. The details of 3-D reconstruction were as follows: First of all, the boundaries of cornea and IOL were detected through a semi-automatic algorithm, which was based on dynamic programming.[17] Secondly, the 3-D data of radical scanning were interpolated and resampled and the surfaces were fitted by the Zernike polynomial.[18] Then the 3-D ray trace was performed in order to correct the optical distortion based on a vector implementation of Snell’s law.[18] After optical distortion correction, the biometry of all parameters was calculated from corrected surfaces. The inner edge of the iris was set manually and fitted by an ellipse based on least squares method. Thus, the orientation and shape of the pupil plane can be obtained. Then the pupil axis was defined as the vector that was perpendicular to the pupil plane and the pupil centre was treated as origin. The IOL axis was then defined as a vector from the anterior IOL centre to the posterior IOL centre. In addition, anterior capsulorhexis was achieved by ellipse fitting from the positions which were extracted from the inner edges of the anterior capsule. The diameter of the anterior capsulorhexis was defined as the average of the short and long axis of the ellipse.(Fig 3)” After 16 images were integrated through semi-auto drawing, 3-D anterior segment of the eye was reconstructed (Fig 4). Finally, the anterior segment parameters were all output automatically (the details are shown in **Table 2**).

**Table 2:** Parameters definition of IOL position after three-dimensional (3-D) construction of anterior segment.

Abbreviated names	Full names	Definition
**PAD**	Postoperative aqueous depth	The distance from central corneal endothelial to the anterior surface of IOL
**IOL-X**	IOL decentration in the x-axis	The displacement of IOL centre relative to the pupil centre in the x-axis
**IOL-Y**	IOL decentration in the y-axis	The displacement of IOL centre relative to the pupil centre in the y-axis
**IOL-D**	IOL decentration in the distance	The absolute distance between the centres of IOL and pupil
**IOL-T**	IOL tilt	The angle of IOL axis and pupil axis

IOL = intraocular lens

### SSOCT imaging

After pupil dilation with 1% tropicamide and 2.5% phenylephrine hydrochloride, all subjects were seated with the headrest and chinrest. Then they were asked to focus on an internal fixation target. In addition, participants were instructed to pull down the lower lid against the lower orbital rim to expose the lower limbus while the technician elevated the upper lid against the upper orbital rim to expose the upper limbus, in order to exclude any lid artifact. Once the subject had been optimally positioned, each eye was scanned with the radial 3-D angle analysis scan using the auto-off function. This high-speed scanning took only 2.4 s and 128 cross-section tomograms of anterior segment were obtained. During the scanning, no eye movement was allowed.

For the measurements repeatability assessment, 3 continuous scans were performed on all eyes. All OCT images were used to rebuild the 3-D anterior segment mode with the new software.

### Parameters

After 3-D reconstruction, the custom-build analysis software is able to output the results of capsule-IOL complex related parameters. **Tables 2–4**showed the details of the parameters.

**Table 3:** Parameters definition of anterior capsulorhexis position after three-dimensional (3-D) construction of anterior segment.

Abbreviated names	Full names	Definition
**Area and D**	Anterior capsulorhexis area and diameter	Anterior capsulorhexis was measured after 3-D construction
**C-X**	Anterior capsulorhexis decentration in the x-axis	The displacement of anterior capsulorhexis centre relative to the pupil centre in the x-axis
**C-Y**	Anterior capsulorhexis decentration in the y-axis	The displacement of anterior capsulorhexis centre relative to the pupil centre in the y-axis
**C-D**	Anterior capsulorhexis decentration in the distance	The absolute distance between the centres of anterior capsulorhexis and pupil

**Table 4:** Parameters definition of anterior capsulorhexis-IOL position after three-dimensional (3-D) construction of anterior segment.

Abbreviated names	Full names	Definition
**C-IOL-X**	Anterior capsulorhexis-IOL decentration in the x-axis	The displacement of anterior capsulorhexis centre relative to the IOL centre in the x-axis
**C-IOL-Y**	Anterior capsulorhexis-IOL decentration in the y-axis	The displacement of anterior capsulorhexis centre relative to the IOL centre in the y-axis
**C-IOL-D**	Anterior capsulorhexis-IOL decentration in the distance	The absolute distance between the centres of anterior capsulorhexis and IOL

IOL = intraocular lens

They are postoperative aqueous depth (PAD)[19–21], IOL decentration in the x-axis (IOL-X), IOL decentration in the y-axis (IOL-Y), IOL decentration in the distance (IOL-D), IOL tilt (IOL-T), anterior capsulorhexis area and diameter (Area and D), anterior capsulorhexis decentration in the x-axis(C-X), anterior capsulorhexis decentration in the y-axis (C-Y), anterior capsulorhexis decentration in the distance (C-D), anterior capsulorhexis-IOL decentration in the x-axis (C-IOL-X), anterior capsulorhexis-IOL decentration in the y-axis (C-IOL-Y), and anterior capsulorhexis-IOL decentration in the distance (C-IOL-D).

### Statistical analysis

Statistical analysis was performed using SPSS software for Windows version 19.0 (SPSS Inc., Chicago, IL, U.S.) and Microsoft Office Excel. To determine the intraobserver repeatability of this new method, mean standard deviation (mean SD), general linear model of repeated measurement, Pearson correlation analysis, and intraclass correlation coefficients (ICCs) were calculated for the three repeated measurements obtained by the same technician. The closer the ICC is to 1, the better the measurement consistency.

Bland-Altman plots[22] were created to assess agreement between three continuous measurements, and the 95% limits of agreement (LoA) were calculated as the mean ± 1.95 SD of the difference. On the Bland-Altman graphs, the difference between each pair of measurements is displayed in the Y-axis, while the mean value in the X-axis. The central line represents the mean of each pair of measurements differences and the broken lines represent the 95% LoA.

## Results

Twenty-two eyes were included in this study and the mean age was 67.8±10.3 years. Measurements of PAD, IOL-T, Area, and D showed high repeatability, as the Mean SD was respectively 0.02 mm, 0.11 mm, 0.22 mm^2^, and 0.03 mm; repeated measures analysis showed there was no statistically significant difference among the three consecutive measurements (all P > .05). Pearson correlation analysis showed high correlation between each pair of them (all r >0.90, P<0.001). ICCs were all more than 0.9 for all parameters. **(Table 5).**

**Table 5:** The repeatability results of PAD, IOL-T, Area, and D using different statistical analysis methods.

Parameters	Mean ± SD	Mean SD	Repeated measurement (P)	Pearson correlation between every two indexes (r, P)	ICC (95%CI)
**PAD**	3.89 mm ± 0.20	0.02	0.440	0.980, <0.001; 0.959,<0.001; 0.975, <0.001	0.971 (0.943–0.987)
**IOL-T**	0.84° ± 0.52	0.11	0.226	0.923, <0.001; 0.931, <0.001; 0.905, <0.001	0.911 (0.831–0.959)
**Area**	19.71mm^2^ ± 3.22	0.22	0.104	0.989, <0.001; 0.995, <0.001; 0.984, <0.001	0.987 (0.975–0.994)
**D**	4.99 mm ± 0.43	0.03	0.125	0.990, <0.001; 0.995, <0.001; 0.985, <0.001	0.989 (0.977–0.995)

PAD = Postoperative aqueous depth; IOL-T = intraocular lens tilt; Area = Anterior capsulorhexis area; D = Anterior capsulorhexis diameter; Mean SD = mean standard deviation; ICC = intraclass correlation coefficients; 95% CI = 95% confidence interval

IOL-X, IOL-Y, IOL-D, C-X, C-Y, C-D, C-IOL-X, C-IOL-Y, and C-IOL-D measurements showed high repeatability, as the Mean SD was between 0.01–0.02 mm; repeated measure analysis showed there was no statistically significant difference among the three continuous measurements (all P > .05). Pearson correlation analysis showed high correlation between each pair of them (all r >0.90, P<0.001). ICCs were all more than 0.9 for all parameters. **(Tables 6–8).**

**Table 6:** The repeatability results of IOL-X, IOL-Y, and IOL-D using different statistical analysis methods.

Parameters	Mean ± SD(mm)	Mean SD	Repeated measurement (P)	Pearson correlation between every two indexes (r, P)	ICC(95%CI)
**IOL-X**	0.08 ± 0.10	0.02	0.635	0.958, <0.001; 0.940, <0.001; 0.947, <0.001	0.947(0.897–0.976)
**IOL-Y**	0.06 ± 0.12	0.01	0.139	0.987, <0.001; 0.970, <0.001; 0.970, <0.001	0.974(0.948–0.988)
**IOL-D**	0.16 ± 0.09	0.02	0.206	0.944, <0.001; 0.943, <0.001; 0.928, <0.001	0.935(0.874–0.970)

IOL = intraocular lens; IOL-X = IOL decentration in the x-axis; IOL-Y = IOL decentration in the y-axis; IOL-D = IOL decentration in the distance; Mean SD = mean standard deviation; ICC = intraclass correlation coefficients; 95% CI = 95% confidence interval

**Table 7:** The repeatability results of C-X, C-Y, C-D using different statistical analysis methods.

Parameters	Mean ± SD(mm)	Mean SD	Repeated measurement (P)	Pearson correlation between every two indexes (r, P)	ICC(95%CI)
**C-X**	0.04 ± 0.13	0.01	0.646	0.974, <0.001; 0.985, <0.001; 0.979, <0.001	0.979(0.959–0.991)
**C-Y**	-0.02± 0.18	0.01	0.749	0.991, <0.001; 0.987, <0.001; 0.983, <0.001	0.987(0.973–0.994)
**C-D**	0.20 ± 0.10	0.02	0.582	0.963, <0.001; 0.964, <0.001; 0.949, <0.001	0.959(0.920–0.982)

C-X = Anterior capsulorhexis decentration in the x-axis; C-Y = Anterior capsulorhexis decentration in the y-axis; C-D = Anterior capsulorhexis decentration in the distance; Mean SD = mean standard deviation; ICC = intraclass correlation coefficients; 95% CI = 95% confidence interval

**Table 8:** The repeatability results of C-IOL-X, C-IOL-Y, C-IOL-D using different statistical analysis methods.

Parameters	Mean ± SD(mm)	Mean SD	Repeated measurement (P)	Pearson correlation between every two indexes (r, P)	ICC(95%CI)
**C-IOL-X**	-0.04±0.16	0.02	0.424	0.982, <0.001; 0.976, <0.001; 0.981, <0.001	0.979(0.958–0.990)
**C-IOL-Y**	-0.07±0.15	0.01	0.241	0.979, <0.001; 0.987, <0.001; 0.973, <0.001	0.979(0.957–0.990)
**C-IOL-D**	0.21± 0.11	0.02	0.361	0.970, <0.001; 0.967, <0.001; 0.961, <0.001	0.964(0.929–0.984)

IOL = intraocular lens; C-IOL-X = Anterior capsulorhexis-IOL decentration in the x-axis; C-IOL-Y = Anterior capsulorhexis-IOL decentration in the y-axis; C-IOL-D = Anterior capsulorhexis-IOL decentration in the distance; Mean SD = mean standard deviation; ICC = intraclass correlation coefficients; 95% CI = 95% confidence interval

**Fig 5:**
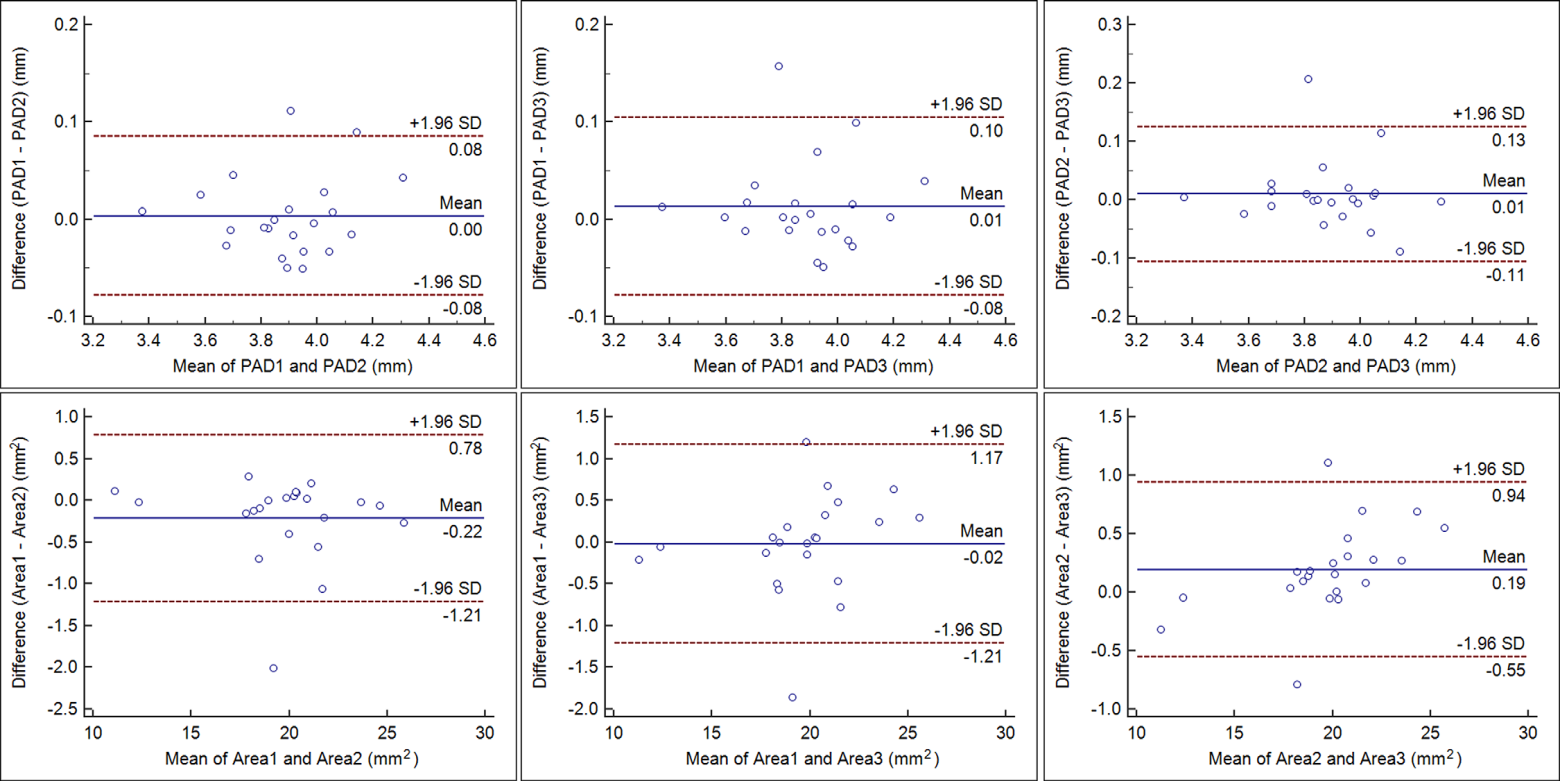
Bland-Altman plots showing three continuous measurements comparisons of postoperative aqueous depth (PAD) and anterior capsulorhexis area (Area). The solid line indicates the mean difference (bias). The upper and lower lines represent the 95% limits of agreement (LoA).

The 95% LoAs of all parameters were narrow for comparision of three measurements, which showed high repeatability for three measurements.(Table 9 and Fig 5).

**Table 9:** The repeatability results of all parameters using Bland-Altman test.

Parameters	95% LoA		
PAD (mm)	-0.08 to 0.08	-0.08 to 0.10	-0.11 to 0.13
IOL-T (°)	-0.41 to 0.55	-0.37 to 0.52	-0.37 to 0.38
Area (mm^2^)	-1.21 to 0.78	-1.21 to 1.17	-0.55 to 0.94
D (mm)	-0.15 to 0.10	-0.16 to 0.15	-0.07 to 0.12
IOL-X(mm)	-0.06 to 0.06	-0.07 to 0.06	-0.07 to 0.06
IOL-Y(mm)	-0.03 to 0.05	-0.05 to 0.07	-0.06 to 0.06
IOL-D(mm)	-0.05 to 0.06	-0.06 to 0.08	-0.05 to 0.07
C-X(mm)	-0.06 to 0.06	-0.05 to 0.06	-0.04 to 0.05
C-Y(mm)	-0.05 to 0.05	-0.07 to 0.07	-0.06 to 0.05
C-D(mm)	-0.05 to 0.06	-0.06 to 0.07	-0.05 to 0.06
C-IOL-X(mm)	-0.06 to 0.06	-0.06 to 0.08	-0.06 to 0.08
C-IOL-Y(mm)	-0.07 to 0.05	-0.08 to 0.06	-0.05 to 0.04
C-IOL-D(mm)	-0.05 to 0.06	-0.06 to 0.07	-0.06 to 0.05

95% LoA = 95% limits of Agreement

PAD = Postoperative aqueous depth; IOL = intraocular lens; IOL-T = intraocular lens tilt

Area = Anterior capsulorhexis area; D = Anterior capsulorhexis diameter;

IOL-X = IOL decentration in the x-axis; IOL-Y = IOL decentration in the y-axis; IOL-D = IOL decentration in the distance

C-X = Anterior capsulorhexis decentration in the x-axis; C-Y = Anterior capsulorhexis decentration in the y-axis; C-D = Anterior capsulorhexis decentration in the distance

C-IOL-X = Anterior capsulorhexis-IOL decentration in the x-axis; C-IOL-Y = Anterior capsulorhexis-IOL decentration in the y-axis; C-IOL-D = Anterior capsulorhexis-IOL decentration in the distance.

## Discussion

The postoperative state of the capsule-IOL complex is very important for the stability of IOL[1, 3, 23, 24] and the occurrence of PCO.[2, 6, 7, 10, 25] However, until now the capsule-IOL complex could not be studied accurately in vivo due to limitations of the imaging techniques.[2, 9, 10, 14] With the method introduced in this study, the capsule-IOL complex could be evaluated and this 3-D method showed a high repeatability of all measured parameters.

Previous studies showed high-quality OCT tomograms of the capsule-IOL complex including the anterior capsule, capsule bending, IOL, posterior capsule, and the space between IOL and posterior capsule.[9–11] However, these studies were all limited on the 2-D level and researchers could only obtain the cross-sectional images along some directions. In this study, 128 high-quality cross-sectional images of the anterior segment were captured with one scan in just 2.4 s. For this new technique, the parameters of capsule-IOL complex could be measured accurately.

Kumar et al.[26] reported the normal in-the-bag IOL maintains an angle with reference to the limbus without causing a significant tilt. Due to the limited scan depth and resolution, the IOL images obtained by Visante OCT were not highly qualified. Besides, only 2-D analysis was available using Visante OCT. Wang et al.[12], Ortiz et al.[27] and Marcos et al.[15] all tried to rebuild the 3-D eye model of anterior segment based on Visante OCT and custom built OCT and evaluated the IOL location. However, no study has mentioned the 3-D reconstruction of postoperative capsule using OCT images. The commercially available SSOCT used in this study provides high-quality images of the capsule-IOL complex for 3-D reconstruction.

Nishi et al.[28] obtained 2 continuous measurements of IOL tilt and decentration in 15 eyes using a clinical Purkinje meter and high repeatability of this method was found (tilt, r = 0.85; decentration, r = 0.95). Wang et al.[12] made 3-D reconstruction based on the Visante OCT images and IOL tilt and decentration were measured 10 times continuously in 3 eyes. General linear model of repeated measurement analysis results showed there was no statistically significant difference in 10 measurements and high repeatability was found (P = 0.351). De Castro et al.[29] studied the repeatability of IOL tilt and decentration measurements using Pentacam and Purkinje meter, respectively. The results showed the mean SD was 0.61 degrees and 0.20 degrees for tilt and 0.05mm and 0.09mm for decentration for Purkinje and Scheimpflug, respectively.

In the present study, we found high repeatability measuring the parameters of capsule-IOL complex using SSOCT. This study firstly assessed the measurements repeatability of capsule related parameters on the 3-D level and high repeatability was found. For IOL tilt and decentration measurements, this new method has much higher repeatability than Pentacam and Purkinje meter. There might be several reasons. Firstly, the commercial SSOCT has high speed of scanning and was easy to capture high-quality images without eye movement. Secondly, the axial resolution is very high (10 um). Third, the measurement accuracy and stability were both improved after the 3-D reconstruction. Similar to some previous studies[12, 14, 29], we selected the pupil axis as the reference. It might be different from the true tilt, but the pupil axis is a constant axis and easy to evaluate.

There are several limitations in this study. Firstly, only one type of IOL was involved and whether this method is suitable for other types of IOLs is still unknown. This would be the next step of our study. Secondly, in the current study, all IOLs were in the bag without any complication. Whether this method is available in the eyes with some complications, such as posterior capsule rupture and ciliary zonules broken and vitreous prolapse, would be demonstrated in the future work. Thirdly, this is not a true 3-D analysis, but a reconstructed model generated from 16 frames. Finally, this study evaluated only the intraobserver repeatability, i.e. only instrumental variations. The reproducibility of interobserver and session variation will be evaluated in the next step of the study.

In conclusion, we showed that the 3-D reconstructed and analysis method, which is based on the SSOCT images, was highly repeatable in measuring the parameters of capsule-IOL complex. Thus, this new method might be the optimal strategy to evaluate the postoperative C-IOL complex and monitor the IOL position and the interaction between the capsule and the IOL. Based on these results, the surgical approach might be optimized to improve the IOL stability, the accuracy of the refractive prediction and reduce PCO.

## Supporting Information

S1 File The study protocol (Chinese and English versions). (RAR)

S2 File The minimal data set underlying the findings of this study. (PDF)
