# Supplementary material for: The Repeatability Assessment of Three-Dimensional Capsule-Intraocular Lens Complex Measurements by Means of High-Speed Swept-Source Optical Coherence Tomography
Source: PLoS One. 2015 Nov 23;10(11):e0142556. doi: 10.1371/journal.pone.0142556 (PMC4658094; doi:10.1371/journal.pone.0142556)
Supplement: S2 File — (PDF) [file pone.0142556.s002.pdf]

NO	PAD	PAD2	PAD3	Area1	Area2	Area3	D1	D2	D3
1	3.597285	3.571565	3.59459	18.06259	17.77212	18.55597	4.799893	4.7611	4.863082
2	4.328736	4.285918	4.289176	18.1026	18.80499	18.66666	4.810209	4.904849	4.880163
3	4.113803	4.129514	4.014671	21.65735	21.86657	21.17134	5.254139	5.277872	5.19607
4	4.058683	4.050662	4.043076	23.63109	23.65458	23.38226	5.491505	5.49309	5.463392
5	3.661446	3.687771	3.672994	18.44699	18.54206	18.44669	4.846983	4.859278	4.847847
6	3.904668	3.920537	3.948752	20.25583	20.20541	20.19789	5.085324	5.078031	5.080339
7	3.378406	3.369801	3.365258	12.32835	12.34329	12.38456	3.968239	3.973286	3.976914
8	3.720974	3.675459	3.685616	20.40314	20.30827	19.19944	5.100884	5.090343	4.946959
9	3.867939	3.917906	3.710263	24.60592	24.66196	23.9711	5.609029	5.610634	5.528623
10	4.025506	4.058817	4.046954	25.70492	25.96598	25.4144	5.726758	5.754203	5.69461
11	4.185939	4.095992	4.183943	21.21849	21.00638	20.54456	5.20042	5.173477	5.116821
12	3.685147	3.695741	3.667503	20.37207	20.26829	20.32594	5.097395	5.083521	5.090937
13	3.903803	3.893388	3.897675	21.16725	22.22642	21.94478	5.192822	5.32562	5.292298
14	3.821174	3.830111	3.831778	19.8463	19.81308	19.86404	5.027259	5.023225	5.030287
15	3.804765	3.81257	3.802282	18.13907	18.26101	18.08413	4.814503	4.832407	4.807387
16	3.959898	3.847886	3.890159	11.17049	11.05833	11.37547	3.771413	3.752619	3.805825
17	3.845831	3.845831	3.845926	18.93807	18.93807	18.75266	4.915528	4.915528	4.891517
18	4.037124	4.008861	4.065151	20.9285	20.90485	20.59766	5.162444	5.161253	5.122311
19	3.921682	3.97214	3.970498	18.18719	20.19603	20.04163	4.814242	5.073416	5.052835
20	3.98447	3.988121	3.994705	17.69193	17.84808	17.81472	4.747038	4.767378	4.764018
21	3.8545	3.894489	3.838063	19.77074	20.16808	19.91448	5.024591	5.070654	5.037019
22	3.934605	3.967443	3.946897	21.17215	21.72146	21.64125	5.19209	5.259159	5.249456

NO	IOL-T1	IOL-T2	IOL-T3	IOL-D1	IOL-D2	IOL-D3	IOL-X1	IOL-X2	IOL-X3
1	1.118481	1.235525	1.291763	0.162067	0.169127	0.162724	0.140498	0.157016	0.133459
2	0.697402	0.516484	0.278356	0.157295	0.103148	0.043168	-0.10443	-0.0529	-0.00228
3	0.40659	0.478896	0.702243	0.070773	0.052	0.066297	0.029301	0.038318	0.052913
4	0.316906	0.310818	0.199963	0.26905	0.216114	0.270639	0.250967	0.210328	0.261066
5	0.096658	0.305621	0.330894	0.163708	0.146369	0.134187	0.161245	0.138584	0.117343
6	1.670485	1.238129	1.255949	0.2811	0.256393	0.287	0.172654	0.148081	0.186137
7	0.582848	0.465239	0.455098	0.158377	0.168164	0.136629	0.14927	0.161941	0.128319
8	0.813813	0.993714	0.77774	0.250945	0.276749	0.263677	-0.10831	-0.07763	-0.10577
9	2.129752	2.196008	1.925427	0.042394	0.046757	0.052175	-0.03015	-0.03495	0.012634
10	1.415341	1.034617	1.309107	0.214041	0.247278	0.18204	0.00547	-0.02706	-0.00549
11	0.876706	1.278027	0.918849	0.191981	0.192686	0.193223	0.114787	0.144358	0.09976
12	0.707755	0.489441	0.644564	0.373598	0.358886	0.360469	-0.06845	-0.10125	-0.0389
13	0.83279	0.555856	0.866279	0.055732	0.073632	0.074527	0.039546	0.035671	0.06761
14	0.47294	0.633475	0.536922	0.18523	0.151291	0.171695	0.054229	0.044906	0.023121
15	1.227752	1.017608	0.719273	0.070859	0.071781	0.060851	0.006568	0.013479	0.00217
16	0.889176	0.780416	0.866971	0.113212	0.137948	0.145675	0.108259	0.136002	0.141723
17	0.114882	0.099503	0.114882	0.107218	0.107218	0.147294	0.092687	0.092687	0.139758
18	0.464278	0.418538	0.637907	0.043271	0.094349	0.035261	0.034004	0.090537	0.02937
19	1.067537	0.434401	0.623793	0.180476	0.12331	0.098098	0.144517	0.072647	0.08213
20	1.212718	1.190272	1.139923	0.277167	0.302881	0.280427	0.271039	0.292932	0.276779
21	2.120812	1.824993	1.872299	0.218099	0.193772	0.19505	0.217891	0.193392	0.194342
22	0.230528	0.428804	0.375653	0.100695	0.082823	0.058682	-0.02112	-0.01422	-0.00319

NO	IOL-Y1	IOL-Y2	IOL-Y3	C-D1	C-D2	C-D3	C-X1	C-X2	C-X3
1	0.080785	0.062847	0.093101	0.32521	0.328249	0.356446	-0.04051	-0.03223	-0.08331
2	0.11763	0.088549	0.043108	0.244942	0.297632	0.287036	0.19807	0.250259	0.242304
3	0.064423	0.035153	0.039944	0.07878	0.103381	0.077389	0.078281	0.099069	0.077386
4	0.096973	0.049673	0.071345	0.194578	0.187713	0.168403	0.055352	0.065983	0.05957
5	-0.02829	-0.0471	-0.06509	0.053411	0.071286	0.0903	-0.01402	-0.01636	-0.01605
6	0.221829	0.209307	0.218454	0.308808	0.22465	0.241919	0.189109	0.146698	0.135305
7	0.052931	0.045322	0.046923	0.113043	0.117852	0.08658	0.085978	0.099596	0.069234
8	-0.22637	-0.26564	-0.24154	0.335923	0.360208	0.349411	-0.01843	-0.02611	-0.02143
9	0.029802	0.031061	0.050623	0.273596	0.260312	0.278836	-0.16034	-0.14972	-0.20639
10	0.213971	0.245793	0.181957	0.01412	0.005309	0.066915	-0.01234	0.001217	0.003727
11	0.153885	0.127628	0.165479	0.251984	0.247756	0.216433	0.180429	0.182669	0.161942
12	0.367273	0.344306	0.358364	0.210957	0.202639	0.187208	-0.01306	-0.0383	-0.00284
13	-0.03927	-0.06442	-0.03136	0.302182	0.300015	0.274682	0.10432	0.174311	0.173018
14	0.177114	0.144473	0.170131	0.257165	0.21954	0.223707	0.252464	0.218417	0.221134
15	-0.07055	-0.0705	-0.06081	0.236042	0.238667	0.173963	0.075405	0.02508	0.046806
16	-0.03312	-0.02309	0.033702	0.068945	0.056015	0.056237	-0.00551	0.001945	0.006151
17	-0.0539	-0.0539	-0.04651	0.249262	0.249262	0.269221	0.135554	0.135554	0.155496
18	0.026759	0.026546	0.019512	0.150855	0.194091	0.154494	0.124256	0.182129	0.137722
19	0.108103	0.099639	0.053647	0.348773	0.367872	0.375663	-0.34734	-0.3676	-0.37354
20	0.057957	0.076992	0.045087	0.128144	0.127454	0.103484	-0.02439	-0.02728	-0.01732
21	-0.00953	0.012133	0.016608	0.294986	0.285534	0.303061	0.159135	0.11575	0.141506
22	0.098456	0.081593	0.058595	0.090791	0.069791	0.057231	-0.04755	-0.05814	-0.04715

NO	C-Y1	C-Y2	C-Y3	C-IOL-D1	C-IOL-D2	C-IOL-D3	C-IOL-X1	C-IOL-X2	C-IOL-X3
1	0.322677	0.326662	0.346573	0.302117	0.324676	0.333524	-0.18101	-0.18925	-0.21677
2	0.144101	0.161106	0.153879	0.303651	0.311723	0.268501	0.302495	0.303161	0.244586
3	-0.00885	-0.02955	-0.00074	0.088133	0.08875	0.047475	0.04898	0.060751	0.024473
4	-0.18654	-0.17573	-0.15751	0.344448	0.267664	0.304921	-0.19562	-0.14435	-0.2015
5	-0.05154	-0.06938	-0.08886	0.176796	0.156541	0.135499	-0.17526	-0.15495	-0.1334
6	0.244131	0.170139	0.200543	0.027716	0.039193	0.053896	0.016455	-0.00138	-0.05083
7	-0.07339	-0.06301	-0.05199	0.141292	0.124988	0.115214	-0.06329	-0.06235	-0.05908
8	-0.33542	-0.35926	-0.34875	0.141317	0.106862	0.13641	0.089882	0.05152	0.084334
9	-0.22169	-0.21295	-0.18749	0.283189	0.269651	0.323526	-0.13019	-0.11477	-0.21902
10	0.006867	-0.00517	-0.06681	0.207869	0.252548	0.248939	-0.01781	0.028279	0.009217
11	-0.1759	-0.16738	-0.14359	0.336255	0.297482	0.315263	0.065641	0.038311	0.062182
12	0.210553	0.198986	0.187187	0.166221	0.158369	0.174935	0.055391	0.062952	0.036065
13	-0.2836	-0.24418	-0.21334	0.252774	0.227018	0.210308	0.064773	0.13864	0.105408
14	0.048947	0.022174	0.033832	0.236059	0.212281	0.240388	0.198235	0.173511	0.198013
15	-0.22367	-0.23735	-0.16755	0.167881	0.167244	0.115693	0.068837	0.011601	0.044636
16	-0.06872	-0.05598	-0.0559	0.119214	0.138033	0.162506	-0.11377	-0.13406	-0.13557
17	-0.20918	-0.20918	-0.21978	0.161092	0.161092	0.173978	0.042866	0.042866	0.015737
18	0.085543	0.067085	0.070008	0.107707	0.100162	0.11954	0.090251	0.091592	0.108352
19	-0.03158	0.014033	-0.03992	0.511307	0.448497	0.465173	-0.49186	-0.44025	-0.45567
20	0.125802	0.124501	0.102024	0.30312	0.323715	0.29956	-0.29543	-0.32021	-0.2941
21	0.24838	0.261021	0.267996	0.264515	0.260716	0.256881	-0.05876	-0.07764	-0.05284
22	0.077346	0.038607	0.032433	0.033825	0.061455	0.051158	-0.02643	-0.04392	-0.04396

NO	C-IOL-Y1	C-IOL-Y2	C-IOL-Y3
1	0.241892	0.263816	0.253472
2	0.026471	0.072557	0.110771
3	-0.07327	-0.0647	-0.04068
4	-0.28351	-0.22541	-0.22886
5	-0.02325	-0.02228	-0.02377
6	0.022302	-0.03917	-0.01791
7	-0.12632	-0.10833	-0.09891
8	-0.10905	-0.09362	-0.10722
9	-0.25149	-0.24401	-0.23811
10	-0.2071	-0.25096	-0.24877
11	-0.32979	-0.295	-0.30907
12	-0.15672	-0.14532	-0.17118
13	-0.24433	-0.17977	-0.18199
14	-0.12817	-0.1223	-0.1363
15	-0.15312	-0.16684	-0.10674
16	-0.03561	-0.03289	-0.0896
17	-0.15528	-0.15528	-0.17327
18	0.058784	0.040538	0.050496
19	-0.13968	-0.08561	-0.09356
20	0.067844	0.047509	0.056938
21	0.257906	0.248887	0.251388
22	-0.02111	-0.04299	-0.02616
